# Supplementary figures and images for: Refinement of efficient encodings of movement in the dorsolateral striatum throughout learning
Source: bioRxiv. 2024 Jun 6:2024.06.06.596654. Preprint. [Version 1] doi: 10.1101/2024.06.06.596654 (PMC11185645; doi:10.1101/2024.06.06.596654)

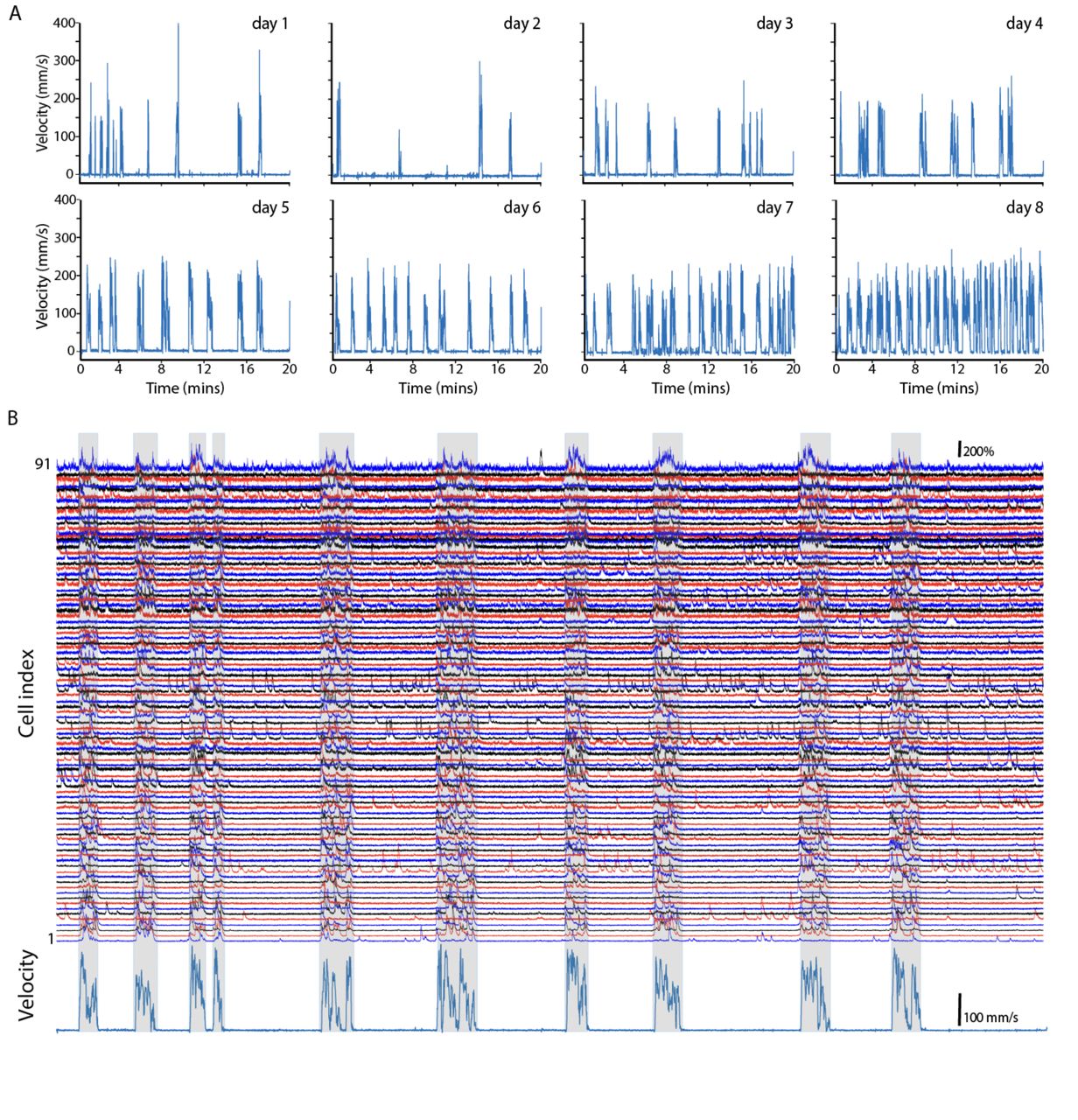

Supplement: Supplement 2 — Figure S2 – Imaging striatal SPNs throughout locomotion paradigm. A, Representative plots of an animal’s velocity across days. B, Representative Ca2+ traces (top) of imaged SPNs as mice perform self-generated bouts of locomotion (bottom). [file figure-S2.jpg]

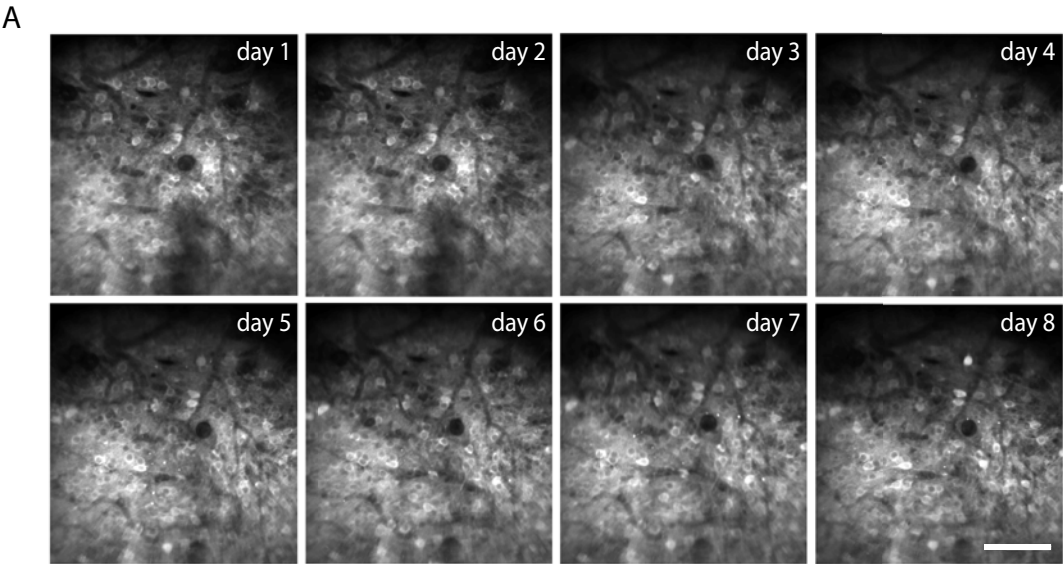

Jáidar et al. - Figure S1

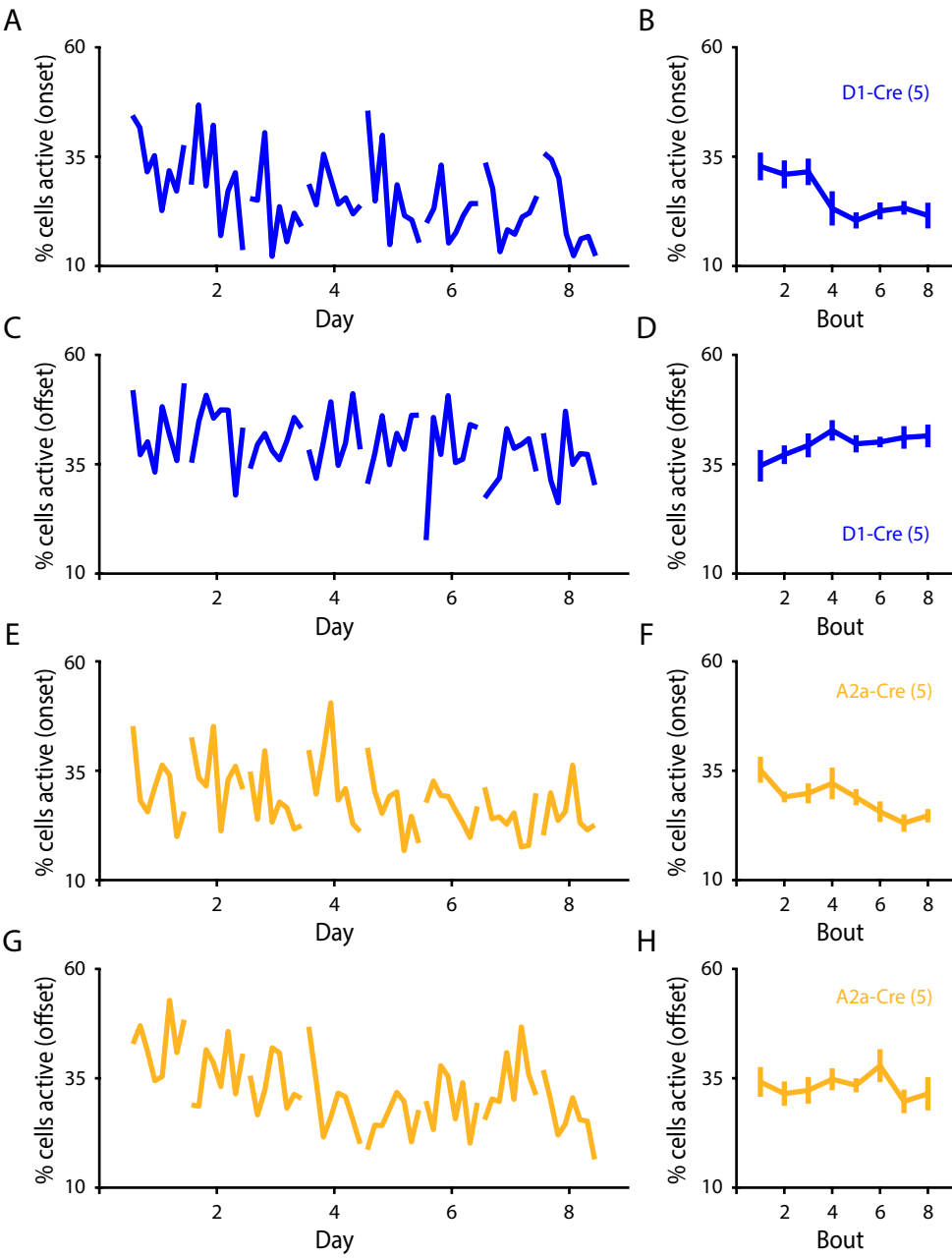

Jáidar et al. - Figure S3

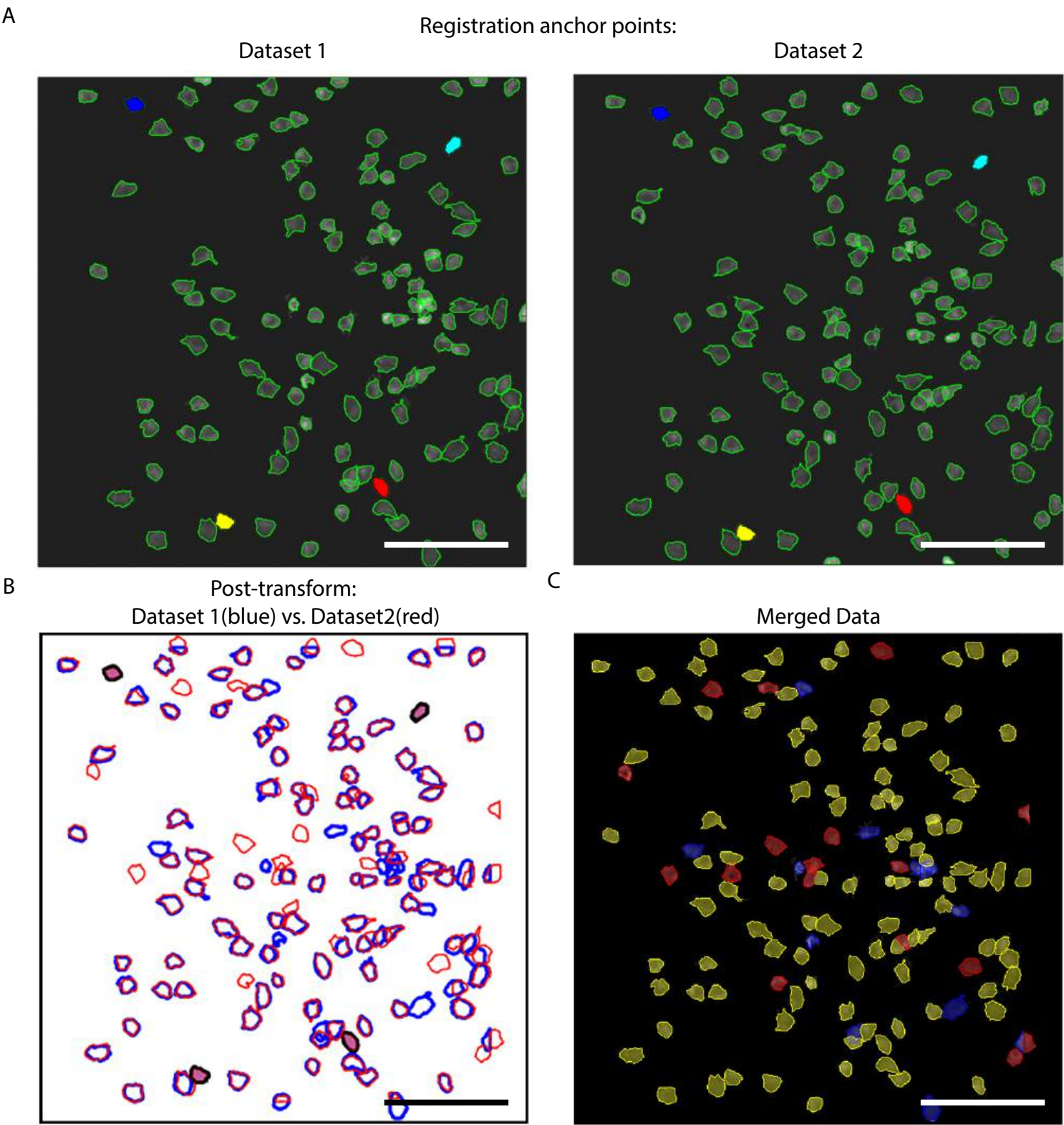

Jáidar et al. - Figure S4

Supplement: Supplement 3 — Figure S1 – Longitudinal imaging of striatal SPNs. A, Representative imaging of the DLS through the 2-photon microscope, depicting consistent revisiting of the same field of view across sessions. Scalebar: 100 μm. Figure S3 – Bout-to-bout decrease in SPN activation during action initiation. A, Percentage of dSPNs active during the first 8 motion onsets of each day (averaged across animals). B, Activation percentages for the first 8 bouts averaged across days revealing a significant decrease in dSPN activation across consecutive motion onsets (n = 5 mice; p = 0.019). C, D, dSPN activation across the initial 8 bouts of motion offsets (n = 5 mice; p = 0.369). E, Percentage of iSPNs active during the first 8 motion onsets of each day (averaged across animals). F, Activation percentages for the first 8 bouts averaged across days revealing a significant decrease in iSPN activation across consecutive motion onsets (n = 5 mice; p = 0.014). G, H, iSPN activation across the initial 8 bouts of motion offsets (n = 5 mice; p = 0.352). Data are mean ± SEM. Statistical significance was assessed by repeated measures 1-way ANOVA with multiple comparisons (B, D, F, H). Figure S4 – Cross-day neuronal alignment. A, Selection of 4 pairs of active neurons shown with different colors in both data sets (dark blue, light blue, yellow, and red filters). B, Post-transformation alignment of both fields of view (FOV). Blue filter contours denote active neurons from data set 1, and red filter contours denote active neurons from data set 2. Filled filters represent the original anchor points used in A.C, Merged FOV image. Blue filters indicate neurons active only in data set 1, red filters indicate neurons active only in data set 2, and yellow filters represent neurons that were active in both data sets. Scale bars: 100μm. [file NIHPP2024.06.06.596654v1-supplement-3.pdf]
